# Altered within- and between-network functional connectivity in atypical Alzheimer’s disease

**DOI:** 10.1093/braincomms/fcad184

**Published:** 2023-06-14

**Authors:** Neha Atulkumar Singh, Peter R Martin, Jonathan Graff-Radford, Irene Sintini, Mary M Machulda, Joseph R Duffy, Jeffrey L Gunter, Hugo Botha, David T Jones, Val J Lowe, Clifford R Jack, Keith A Josephs, Jennifer L Whitwell

**Affiliations:** Department of Neurology, Mayo Clinic, Rochester, MN 55905, USA; Department of Quantitative Health Sciences, Mayo Clinic, Rochester, MN 55905, USA; Department of Neurology, Mayo Clinic, Rochester, MN 55905, USA; Department of Radiology, Mayo Clinic, Rochester, MN 55905, USA; Department of Psychiatry & Psychology, Mayo Clinic, Rochester, MN 55905, USA; Department of Neurology, Mayo Clinic, Rochester, MN 55905, USA; Department of Radiology, Mayo Clinic, Rochester, MN 55905, USA; Department of Neurology, Mayo Clinic, Rochester, MN 55905, USA; Department of Neurology, Mayo Clinic, Rochester, MN 55905, USA; Department of Radiology, Mayo Clinic, Rochester, MN 55905, USA; Department of Radiology, Mayo Clinic, Rochester, MN 55905, USA; Department of Neurology, Mayo Clinic, Rochester, MN 55905, USA; Department of Radiology, Mayo Clinic, Rochester, MN 55905, USA

**Keywords:** resting-state functional connectivity, logopenic progressive aphasia, posterior cortical atrophy, atypical Alzheimer’s disease, brain network disruptions

## Abstract

Posterior cortical atrophy and logopenic progressive aphasia are atypical clinical presentations of Alzheimer’s disease. Resting-state functional connectivity studies have shown functional network disruptions in both phenotypes, particularly involving the language network in logopenic progressive aphasia and the visual network in posterior cortical atrophy. However, little is known about how connectivity differs both within and between brain networks in these atypical Alzheimer’s disease phenotypes. A cohort of 144 patients was recruited by the Neurodegenerative Research Group at Mayo Clinic, Rochester, MN, USA, and underwent structural and resting-state functional MRI. Spatially preprocessed data were analysed to explore the default mode network and the salience, sensorimotor, language, visual and memory networks. The data were analysed at the voxel and network levels. Bayesian hierarchical linear models adjusted for age and sex were used to analyse within- and between-network connectivity. Reduced within-network connectivity was observed in the language network in both phenotypes, with stronger evidence of reductions in logopenic progressive aphasia compared to controls. Only posterior cortical atrophy showed reduced within-network connectivity in the visual network compared to controls. Both phenotypes showed reduced within-network connectivity in the default mode and sensorimotor networks. No significant change was noted in the memory network, but a slight increase in the salience within-network connectivity was seen in both phenotypes compared to controls. Between-network analysis in posterior cortical atrophy showed evidence of reduced visual-to-language network connectivity, with reduced visual-to-salience network connectivity, compared to controls. An increase in visual-to-default mode network connectivity was noted in posterior cortical atrophy compared to controls. Between-network analysis in logopenic progressive aphasia showed evidence of reduced language-to-visual network connectivity and an increase in language-to-salience network connectivity compared to controls. Findings from the voxel-level and network-level analysis were in line with the Bayesian hierarchical linear model analysis, showing reduced connectivity in the dominant network based on diagnosis and more crosstalk between networks in general compared to controls. The atypical Alzheimer’s disease phenotypes were associated with disruptions in connectivity, both within and between brain networks. Phenotype-specific differences in connectivity patterns were noted in the visual network for posterior cortical atrophy and the language network for logopenic progressive aphasia.

## Teaser

Singh *et al.* report functional connectivity disruptions in atypical Alzheimer’s disease phenotypes. Phenotype-specific reductions were noted within dominant networks, visual for posterior cortical atrophy and language for logopenic progressive aphasia, with evidence of heightened crosstalk between networks, suggesting possible brain network reorganization following impairment of phenotype-specific dominant networks.

## Graphical Abstract

**Graphical Abstract:**
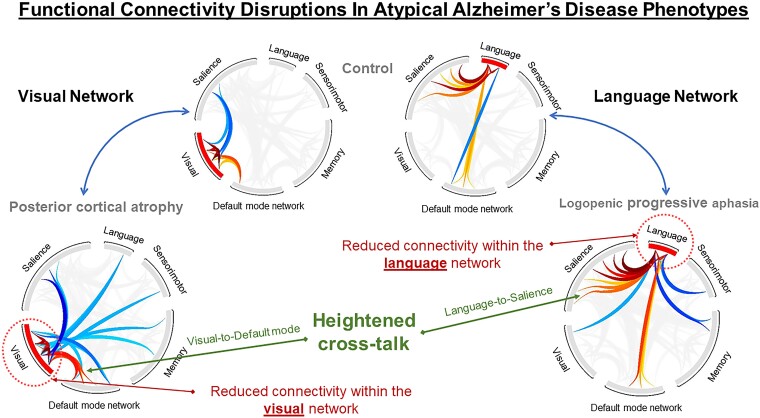

## Introduction

Atypical non-amnestic presentations of Alzheimer’s disease (AD) are typically associated with young onset and can be characterized by the presence of initial and dominant visual, language, behavioural, executive or motor difficulties.^1–4^ Two widely studied phenotypes include posterior cortical atrophy (PCA) and logopenic progressive aphasia (LPA).^5–7^

PCA is characterized by prominent visuospatial and visuoperceptual deficits, with features including simultanagnosia, Gerstmann’s syndrome, constructional dyspraxia, environmental agnosia, oculomotor apraxia, dressing apraxia, optic ataxia and homonymous visual field defects.^8^ PCA patients generally have good insight into their symptoms^9,10^ and relatively preserved episodic memory, behaviour and cognition earlier in the disease.^8^ Language becomes impaired in the later stages of the disease.^11,12^ Patterns of atrophy and hypometabolism on [^18^F]fluorodeoxyglucose PET are predominantly in the visual association areas, mainly the occipito-parietal cortices.^13–15^ LPA is characterized by primary language deficits such as anomia, sentence repetition deficits and phonological errors, with preserved word comprehension, grammar and semantic knowledge.^7^ Patterns of atrophy and hypometabolism on [^18^F]fluorodeoxyglucose PET are predominantly in the left lateral temporal and inferior parietal regions.^16,17^

Resting-state functional MRI (rsfMRI) is widely used to measure blood-oxygen-level-dependent changes in order to examine brain networks of patients at rest.^18^ rsfMRI studies have identified several large-scale functional networks showing correlated and anticorrelated activity fluctuations between neighbouring and distant brain regions.^19^ Two of the most investigated core networks include the default mode network (DMN) and the salience network. The DMN is a specific network of brain regions that become engaged during random episodic silent thinking, i.e. unconstrained thoughts (self-generated thoughts) as opposed to more focused episodic memory activity,^20^ and is involved in cognitive processing.^21^ The salience network is critical for social–emotional functioning,^22^ communication, self-awareness and some aspects of cognition.^23^ Alterations in these functional networks have been reported in typical AD patients, specifically reduced functional connectivity in the DMN and increased functional connectivity in the salience network.^24–26^ Other networks of interest in atypical AD include the visual,^27^ language^28^ and memory^29^ networks. Studies with typical amnestic AD patients have reported associations between loss of functional connectivity and memory loss^30^ and reductions in within-network connectivity in the memory network.^31^ Some studies with a small pool of PCA and LPA patients have also reported disruptions in visual network connectivity for PCA^27,32^ and trends for altered language network connectivity in LPA,^28,32^ but findings for the DMN have been inconsistent in both PCA^27,32^ and LPA.^32^ Little is known about how functional connectivity profiles differ between LPA and PCA and how these syndromes alter connectivity between networks.

The aim of our study was to assess functional connectivity profiles in a large cohort of patients clinically diagnosed as PCA and LPA and evaluate how within- and between-network functional connectivity differs between the two phenotypes and from controls. We hypothesized reduced within-network connectivity in the visual network for PCA and the language network for LPA, along with an increase in between-network connectivity, which would be indicative of crosstalk or brain network rewiring. As a secondary aim, we will also carry out a sensitivity analysis to compare functional connectivity measures to other neuroimaging measures, specifically grey matter volume and tau-PET uptake, to investigate which better mirrors clinical syndrome.

## Materials and methods

### Patients

Fifty-nine patients that fulfilled clinical diagnostic criteria for PCA^8^ and 85 patients that fulfilled clinical diagnostic criteria for LPA^6,7^ were recruited by the Neurodegenerative Research Group from the Department of Neurology, Mayo Clinic, Rochester, MN, USA, between 29 November 2010 and 28 February 2020 into an NIH-funded grant. All patients were enrolled into this study regardless of age and underwent extensive neurological evaluations by one of two behavioural neurologists (K.A.J. or J.G.-R.) and neuropsychological testing overseen by a neuropsychologist (M.M.M.). All patients also completed a structural head MRI scan that included a rsfMRI protocol and a Pittsburgh Compound B PET scan for β-amyloid detection. All 144 patients in this study showed evidence of β-amyloid deposition on PET. All clinical diagnoses were rendered blinded to imaging results. A cohort of 50 cognitively normal healthy amyloid-negative individuals was recruited by the Alzheimer’s Disease Neuroimaging Initiative (ADNI) between 1 January 2017 and 30 August 2021 and underwent identical MRI scans. Ethics approval was obtained by the ADNI investigators.

### Patient consent and protocols

The study was approved by the Mayo Clinic IRB. All patients gave written informed consent to participate in this study.

### Clinical testing

The neurological evaluations of the PCA and LPA patients included the Montreal Cognitive Assessment Battery (MoCA) for assessing general cognitive function,^33^ the Movement Disorders Society–sponsored revision of the Unified Parkinson’s disease rating scale III (UPDRS III) to assess for parkinsonism^34^ and the Neuropsychiatric Inventory test to assess for neuropsychiatric features.^35^ A battery of tests for simultanagnosia was also administered, which included (i) Ishihara colour plates, (ii) images of overlapping line drawings, (iii) colour images of complex picture scenes, and (iv) Navon figures, with performance scored on a 20-point scale (scores under 17 were considered abnormal based on performance in normal controls).^36^ The neuropsychological evaluation included the 15-item Boston Naming Test (BNT) to assess confrontation naming,^37^ Boston Diagnostic Aphasia Exam (BDAE) repetition subtest to assess sentence repetition,^38^ Rey Auditory Verbal Learning Test—Recognition Percent Correct (AVLT-RPC) to measure episodic memory,^39^ facial recognition test for evidence of prosopagnosia,^40^ Rey-Osterrieth Complex Figure Test (Rey-O) to assess visuospatial constructional ability,^41^ Visual Object and Space Perception Battery (VOSP) cubes to assess visuospatial ability and VOSP letters for assessing visuoperceptual ability.^42^

### Image acquisition

All patients underwent scanning with a 3T volumetric MRI on GE scanners (GE Healthcare, Milwaukee, WI, USA) at Mayo Clinic, Rochester, MN, USA, which included a magnetization-prepared rapid gradient echo (MPRAGE) sequence [repetition time (TR)/echo time (TE)/T_1_ = 2300/3/900 ms; 26 cm field of view, slice thickness = 1.2 mm, in-plane resolution = 1 mm],^43^ and rsfMRI scanning using gradient echo planer imaging (TE = 30 ms; slice thickness = 3.3 mm, in-plane resolution = 3.3 mm and 160 volumes). Participants were instructed to keep their eyes open during the scan.^44^

The tau-PET scans were acquired on PET/CT scanners. Patients were injected with ∼370 MBq (range 333–407 MBq) of [^18^F]flortaucipir. Eighty minutes after injection, PET acquisition was carried out for 20 min. The scans consisted of four 5 min dynamic frames after a low-dose CT image. After standard corrections, PET sinograms were reconstructed into a 256 mm field of view. The four individual frames were averaged for analysis.^45^

### Image processing

All fMRI images were preprocessed using the CONN functional connectivity toolbox^46^ (www.nitrc.org/projects/conn). The preprocessing discarded the first 10 volumes to generate steady-state magnetization, slice time correction, realignment (motion estimation and correction), with outlier detection, segmentation and direct normalization to MNI template space, smoothing with a Gaussian kernel of 6 mm full width, half maximum, nuisance regression for white matter, CSF signals, denoised for six head motion parameters with their first- and second-order derivatives,^47,48^ and bandpass filtered in the 0.01–0.1 Hz frequency to reduce low frequency drift and noise effects.^49^ After preprocessing, the functional images were parcellated with the Harvard-Oxford atlas. The mean blood-oxygen-level-dependent time series within each region of interest (ROI) of the atlas was extracted. Pearson’s *R* correlation coefficients were calculated across all ROIs and were transformed to Fisher’s *R*-to-*Z* transformations. Voxel-level (ROI-to-voxel) and network-level (ROI-to-ROI) connectivity maps were generated for six networks. Each network consisted of multiple ROIs, where the functional connectivity of a network was generated by averaging the signal across multiple ROIs within that network. The six networks are as follows: the visual (medial primary visual cortex, lateral primary visual cortex and lateral visual association area), language (left inferior frontal cortex and posterior superior temporal gyrus), DMN (posterior parietal cortex, medial prefrontal cortex and lateral parietal cortex), memory (amygdala, hippocampus and anterior parahippocampal gyrus), sensorimotor (primary motor area and primary sensory area) and salience (rostral prefrontal cortex, anterior cingulate cortex, supramarginal gyrus and insula) networks. These networks were chosen as they have been associated with the cognitive functioning^24,25^ and the dominant aspects of PCA^27^ and LPA.^28^

The tau-PET images were registered to their corresponding subject-space MPRAGE T_1_-weighted MRI using SPM12. Regional PET values were calculated using advanced normalization tools (ANTs)^50^ to propagate the Harvard-Oxford atlas. Unified segmentation^51^ determined tissue probabilities of each MPRAGE scan.^52^ Median tau-PET values were calculated across grey and white matter and divided by the cerebellar crus grey matter median uptake value to generate standardized uptake value ratios. Grey matter volumes were calculated and normalized to the Harvard-Oxford atlas. Grey matter volume and tau-PET uptake composites for each network were created using the same atlas employed in the rsfMRI analysis, i.e. the Harvard-Oxford atlas.

### Statistical analysis

#### Bayesian hierarchical linear models

Bayesian hierarchical linear models (BHLMs) were used to assess within- and between-network connectivity across PCA, LPA and healthy control groups. More specifically, the first BHLM used within-network functional connectivity as the outcome predicted by (in each network), fixed effects for an intercept per group, an age adjustment per group and a sex adjustment per group. This model also included a random intercept per person to account for the multiple network measures per person in this single model. The second and third models used the same formulation, only changing the outcome to between-network functional connectivity from the visual network to all other networks using PCA and healthy control cases and then between-network functional connectivity from the language network to all other networks using LPA and healthy control cases.

Lastly, we fit a fourth BHLM to assess associations with clinical performance by predicting log-transformed within-network functional connectivity as the outcome using, in each network, fixed effects for an overall intercept, an association with each *z*-scored clinical test and terms for age, sex and education per network. These models also included a random intercept per person. The data in this final model included DMN and visual within-network functional connectivity and VOSP letters, VOSP cubes and simultanagnosia from PCA individuals (visual-based clinical tests associated with PCA visual and DMN networks) and salience and language within-network functional connectivity and BDAE repetition and BNT from LPA individuals (language-based clinical tests associated with LPA salience and language networks). Here, the visual network in PCA and the language network in LPA represent the dominant networks for phenotypes.^53^ The DMN, which includes the posterior parietal cortex, a region known to be affected in PCA, and the salience network, which showed higher within-network connectivity in LPA, were also studied. From this model, we adjusted for covariates of age, sex and education to quantify relationships between performance on clinical measures and within-network connectivity.

BHLMs are well suited to analyse neuroimaging data when there are multiple measurements per scan per person. Hierarchical models decrease the total number of models used, incorporate shrinkage into parameter estimates and use partial pooling to increase the precision of fixed effects in the model, all of which reduce the chance of Type I error (i.e. proactively addressing the problem of multiple comparisons), while also allowing for many direct statistical comparisons from a single model.^54–57^ These models were fit using the statistical software R^58^ version 4.1.2 using the rstanarm^59,60^ package version 2.21.1 in conjunction with STAN version 2.21.0. These posterior samples provided distributions for each model parameter, and we then summarized these distributions using probabilities. We assessed the proportion of the distribution that was greater than zero and compared the proportion of samples from one estimate that was greater than a second estimate. The results of these comparisons were reported as posterior probabilities, denoted as posterior probability (Pb) in this paper, and we considered Pb > 0.90 or <0.10 as moderate evidence of a difference and Pb > 0.99 or <0.01 as strong evidence of a difference. Here, we use the term moderate and strong evidence as ways to tier the significance of our results. We believe that moderate evidence, i.e. 9 out of every 10 moderate results are ‘true’ results, will absolutely help guide future research. If we report 10 regions of the brain that are different between two disease groups, the long-run average would say that 9 of those are likely ‘true’ differences, and thus, it seems very helpful for other researchers and future studies to explore those regions as potentially important biomarkers in other cohorts.

#### Voxel- and network-level analysis

The CONN functional connectivity toolbox^46^ (www.nitrc.org/projects/conn) was used to generate voxel-level and network-level maps for each network within each group: PCA, LPA and controls. Group-averaged connectivity maps (one-sample *t*-test) were corrected for a false discovery rate (FDR) at *P* < 0.001, and the difference maps (two-sample *t*-test) were uncorrected at *P* < 0.01 at both the voxel level, with cluster size corrected for family-wise error (FWE) at *P* < 0.05, and the network level, with cluster size corrected for FDR at *P* < 0.05.

#### Sensitivity analysis

To investigate the discriminatory power of functional connectivity compared to grey matter volume and tau-PET uptake, areas under the receiver operating characteristic curves were calculated to provide an effect size measure. Linear regression models were also fit to evaluate the relationship between clinical tests and grey matter volume and tau-PET uptake.

## Results

### Study demographics

The demographic and clinical features of the cohorts are shown in Table 1. There was no evidence of PCA and LPA differing in sex and education, but there were significant differences in age at onset and disease duration, with younger age at onset and longer disease duration in PCA. On clinical testing, PCA performed worse than LPA on simultanagnosia (*P* < 0.001), Rey-O complex figure (*P* < 0.001), facial recognition (*P* = 0.002), VOSP cubes (*P* < 0.001) and VOSP letters (*P* < 0.001), while LPA performed worse than PCA on BNT (*P* < 0.001) and BDAE repetition (*P* = 0.007).

**Table 1:** Participant’s demographics and disease characteristics

	Disease cohort (*N* = 144)			Healthy controls (*N* = 50)
	PCA (*N* = 59)	LPA (*N* = 85)	*P*-value	
Female, *n* (%)	36 (61%)	48 (56.5%)	0.586	29 (58%)
Education, years	16 (13, 16)	16 (14, 16)	0.731	16 (15, 18)
Age at onset, years	59.6 (54.1, 63.4)	64.8 (58.2, 69.2)	<0.001	−
Age at scan, years	63.5 (58.5, 69.4)	67.9 (61.5, 73.2)	0.021	68.2 (65.7, 70.4)
Disease duration, years	4.1 (3.1, 5.6)	3.0 (2.1, 4.3)	0.002	−
MoCA (30)	18 (12, 21)	18 (11, 21)	0.355	28 (26, 30)
BNT (15)	12 (10, 14)	9 (5, 12)	<0.001	−
BDAE repetition (10)	8 (6, 10)	7 (5, 8)	0.007	−
Rey-O MOANS (18)	2 (2, 2)	6 (2, 10)	<0.001	−
VOSP cubes (10)	2 (0, 4.8)	9 (5, 10)	<0.001	−
VOSP letters (20)	12 (6, 16)	19 (18, 20)	<0.001	−
AVLT-RPC (100)	68.3 (60.8, 83.3)	73.3 (63.3, 83.3)	0.766	−
Simultanagnosia (20)	8 (3, 12)	19 (17, 20)	<0.001	−
Famous faces, recognition (10)	10 (7, 10)	10 (9, 10)	0.002	−
UPDRS III (132)	2 (0, 6)	4 (1, 7)	0.227	−
NPI-Q (36)	3 (1, 6)	2 (1, 4)	0.027	−

Data shown are *n* (%) or median (first and third quartiles). For continuous variables, *P*-values are from the Wilcoxon rank sum test. For categorical variables, *P*-values are from Fisher’s exact test. Abbreviations: AVLT-RPC = Auditory Verbal Learning Test—Recognition Percent Correct; BDAE = Boston Diagnostic Aphasia Exam; BNT = Boston Naming Test; LPA = logopenic progressive aphasia; MOANS = Mayo Older Americans Normative Studies; MoCA = Montreal Cognitive Assessment Battery; NPI-Q = Neuro-Psychiatric Inventory Questionnaire; PCA = posterior cortical atrophy; Rey-O = Rey-Osterrieth Complex Figure Test; UPDRS III = Unified Parkinson’s disease rating scale III; VOSP = Visual Object and Space Perception Battery.

### Reduced within-network connectivity in posterior cortical atrophy and logopenic progressive aphasia

#### Visual network

**Figure 1:**
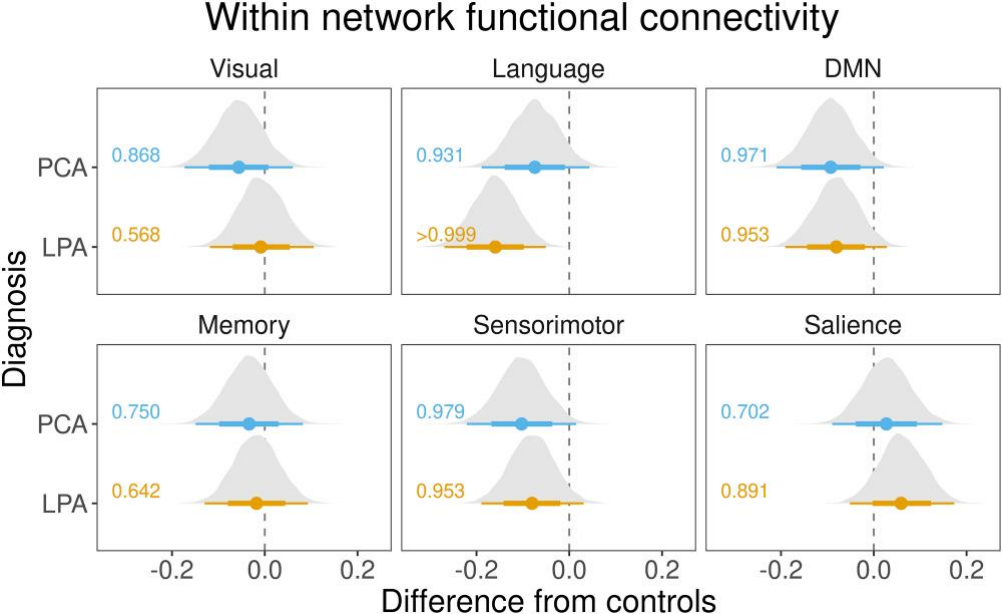
**Within-network functional connectivity in PCA and LPA from healthy controls.** These forest plots compare within-network connectivity for the six major networks: DMN and visual, language, memory, salience and sensorimotor networks in PCA and LPA from controls. The plot shows estimates (median), 80% posterior interval (thick line) and 98% posterior interval (thin line). It is interpreted as follows: when the 80% posterior interval does not touch zero, we say there is moderate evidence (Pb > 0.90) of difference, and when the 98% posterior interval does not touch zero, we say there is strong evidence (Pb > 0.99) of difference from controls.

**Figure 2:**
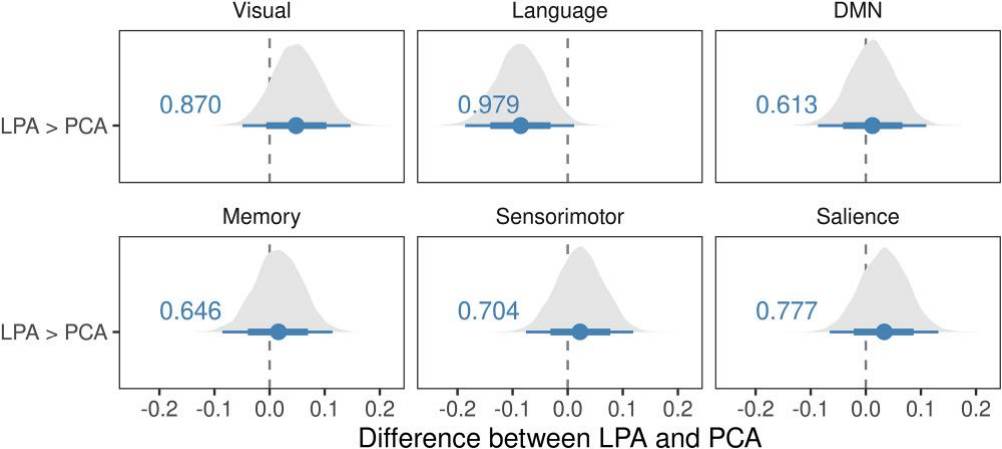
**Within-network functional connectivity comparison between PCA and LPA.** These forest plots compare within-network connectivity differences for the six major networks: DMN and visual, language, memory, salience and sensorimotor networks in PCA and LPA. The plot shows estimates (median), 80% posterior interval (thick line) and 98% posterior interval (thin line). It is interpreted as follows: when the 80% posterior interval does not touch zero, we say there is moderate evidence (Pb > 0.90) of difference, and when the 98% posterior interval does not touch zero, we say there is strong evidence (Pb > 0.99) of difference from controls.

**Figure 3:**
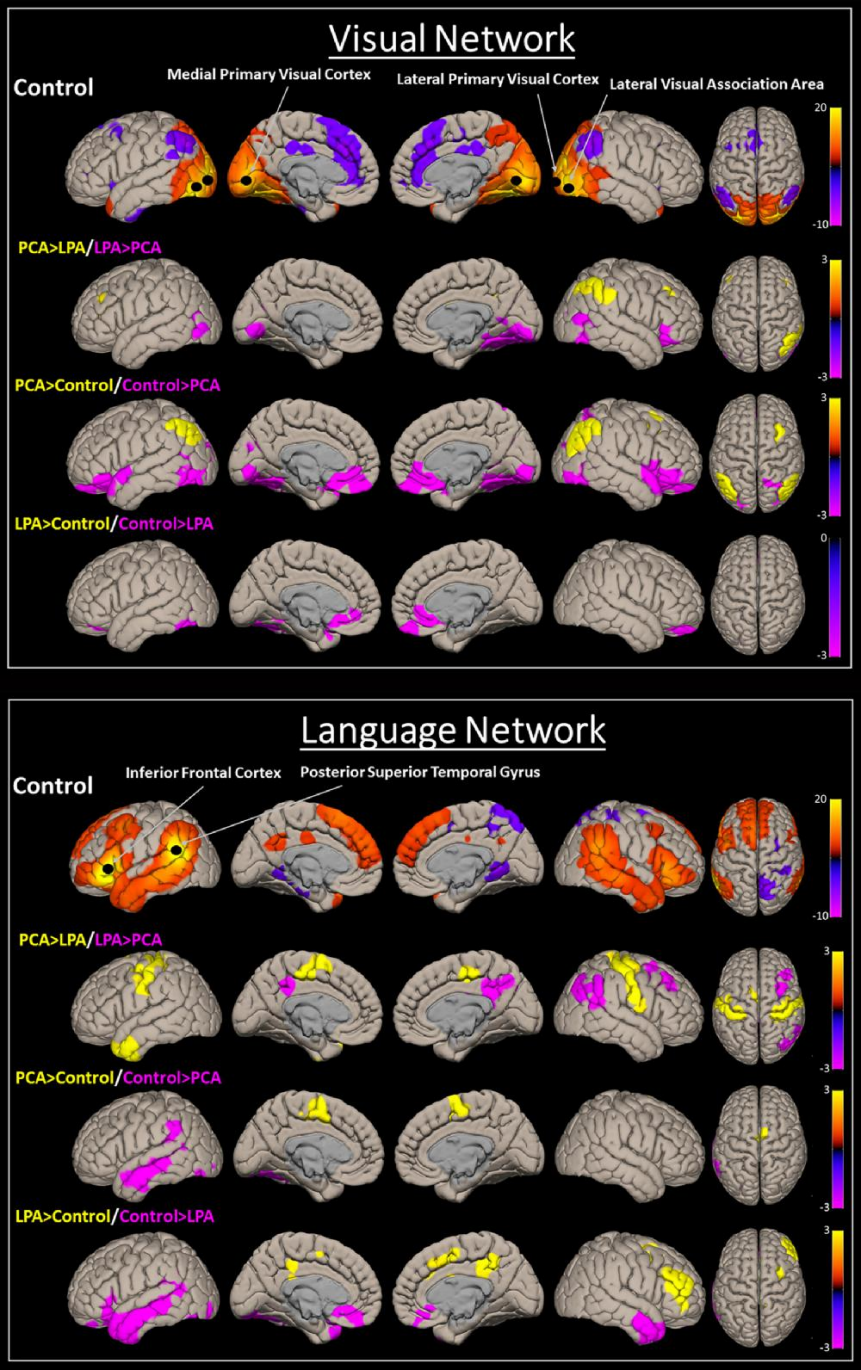
**Functional connectivity voxel-level maps.** Multiple slices of group-level (*P* < 0.001, FDR; cluster size = *P* < 0.05, FWE) functional connectivity patterns for the visual and language networks are shown here for controls, along with their difference (*P* < 0.01; cluster size = *P* < 0.05, FWE) maps between PCA and LPA and from controls. The plot shows *t*-scores generated from one-sample and two-sample *t*-tests.

The BHLM showed weak evidence of reduced visual network connectivity in PCA compared to controls (Pb > 0.868) and LPA (Pb > 0.870) (Figs 1 and 2). No significant differences were noted in LPA (Fig. 1). At the voxel level, PCA showed reduced connectivity in the occipital lobe and increased connectivity in the parietal lobe compared to controls and LPA (Fig. 3).

#### Language network

The BHLM showed evidence of reduced language network connectivity in LPA (Pb > 0.999) and PCA (Pb > 0.931) compared to controls (Fig. 1), with LPA (Pb > 0.979) showing reduced language network connectivity compared to PCA as well (Fig. 2). At the voxel level, both PCA and LPA showed reduced connectivity in the left temporal lobe, worse in LPA, compared to controls. PCA also showed reduced right parietal lobe connectivity compared to controls and LPA. LPA showed reduced sensorimotor connectivity compared to PCA (Fig. 3).

#### Default mode network

**Figure 4:**
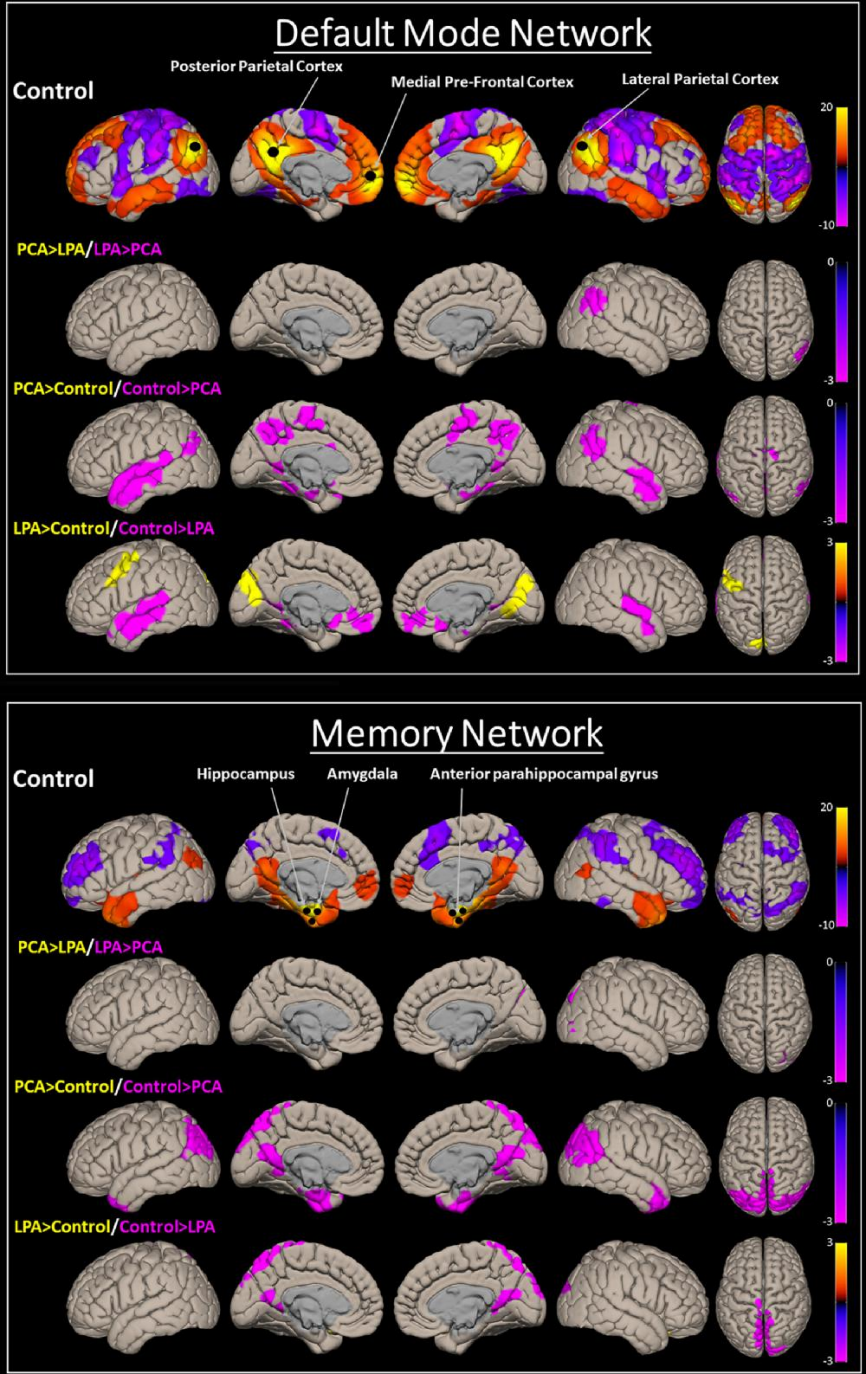
**Functional connectivity voxel-level maps.** Multiple slices of group-level (*P* < 0.001, FDR; cluster size = *P* < 0.05, FWE) functional connectivity patterns for the DMN and memory network are shown here for controls, along with their difference (*P* < 0.01; cluster size = *P* < 0.05, FWE) maps between PCA and LPA and from controls. The plot shows *t*-scores generated from one-sample and two-sample *t*-tests.

The BHLM showed evidence of reduced DMN network connectivity in both PCA (Pb > 0.971) and LPA (Pb > 0.953) compared to controls (Fig. 1). No significant differences were noted between PCA and LPA (Fig. 2). At the voxel level, both PCA and LPA showed reduced temporal lobe connectivity, with LPA showing increased occipital lobe connectivity compared to controls. PCA showed reduced parietal lobe connectivity compared to LPA (Fig. 4).

#### Memory network

The BHLM did not show any significant within-network changes in PCA and LPA compared to controls (Fig. 1) and each other (Fig. 2). At the voxel level, both PCA and LPA showed reduced parietal lobe connectivity, with PCA showing reduced connectivity in the occipital lobe, compared to controls, with little difference observed between PCA and LPA (Fig. 4).

#### Sensorimotor network

**Figure 5:**
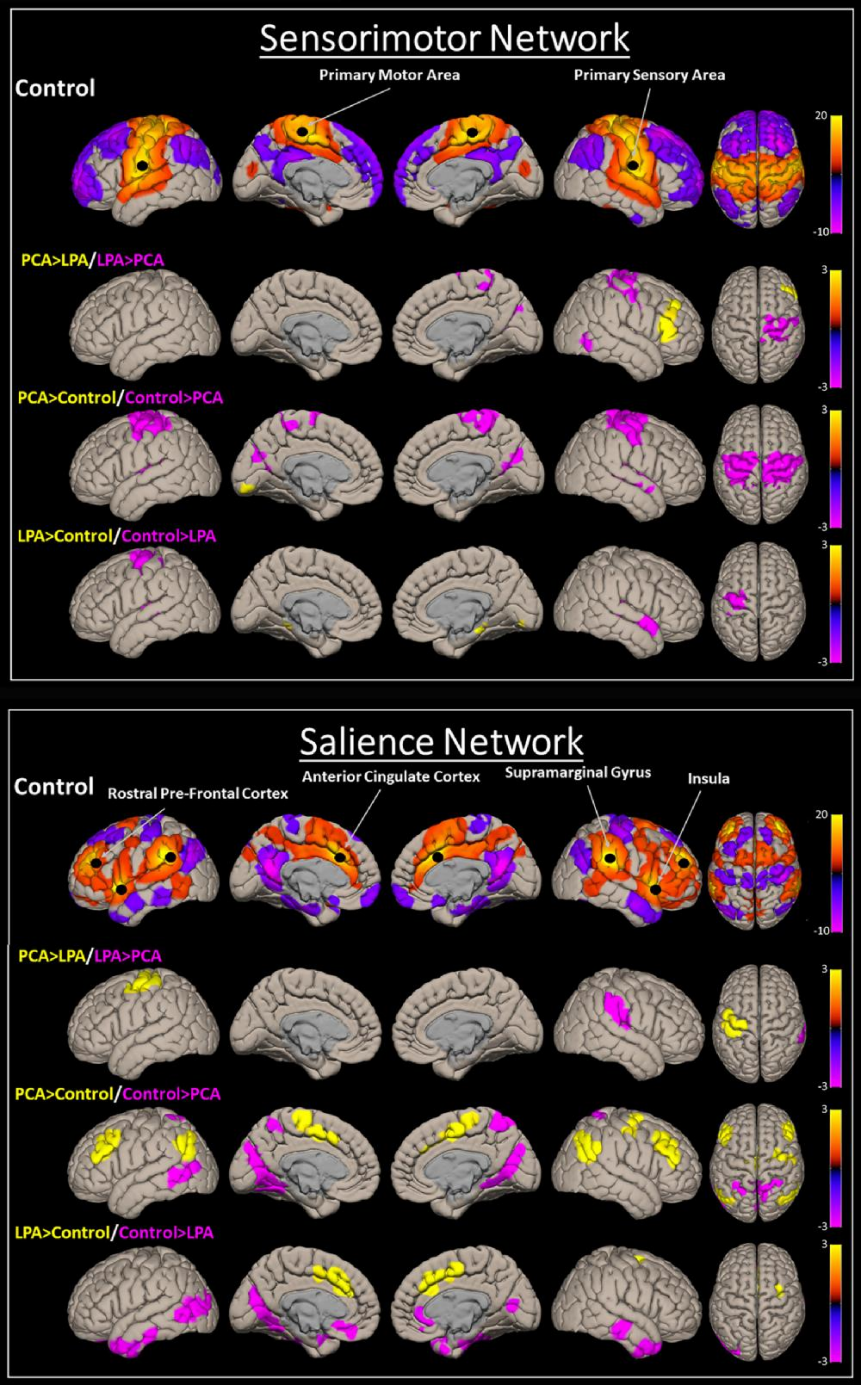
**Functional connectivity voxel-level maps.** Multiple slices of group-level (*P* < 0.001, FDR; cluster size = *P* < 0.05, FWE) functional connectivity patterns for the sensorimotor and salience networks are shown here for controls, along with their difference (*P* < 0.01; cluster size = *P* < 0.05, FWE) maps between PCA and LPA and from controls. The plot shows *t*-scores generated from one-sample and two-sample *t*-tests.

The BHLM showed evidence of reduced sensorimotor network connectivity in PCA (Pb > 0.979) and LPA (Pb > 0.953) compared to controls (Fig. 1). No significant differences were noted between PCA and LPA (Fig. 2). At the voxel level, both PCA and LPA showed reduced connectivity in the sensorimotor cortices, with bilateral reductions in PCA and left in LPA, compared to controls. PCA showed reduced right sensorimotor connectivity compared to LPA, and LPA showed reduced right frontal lobe connectivity compared to PCA (Fig. 5).

#### Salience network

The BHLM showed weak evidence of increased salience network connectivity in LPA (Pb > 0.891) compared to controls. No significant differences were noted in PCA compared to controls (Fig. 1). Moreover, no significant differences were noted when comparing PCA to LPA (Fig. 2). At the voxel level, PCA showed increased connectivity in the dorsolateral frontal lobe, posterior medial frontal lobe and inferior parietal lobe compared to controls, with increased connectivity in the sensorimotor cortex compared to LPA. LPA showed increased connectivity in the posterior medial frontal lobe compared to controls. Both PCA and LPA showed reduced connectivity in the temporal and occipital lobes compared to controls (Fig. 5). The effect of adjustment factors is shown in Supplementary Fig. 1.

### Altered between-network connectivity in posterior cortical atrophy and logopenic progressive aphasia

**Figure 6:**
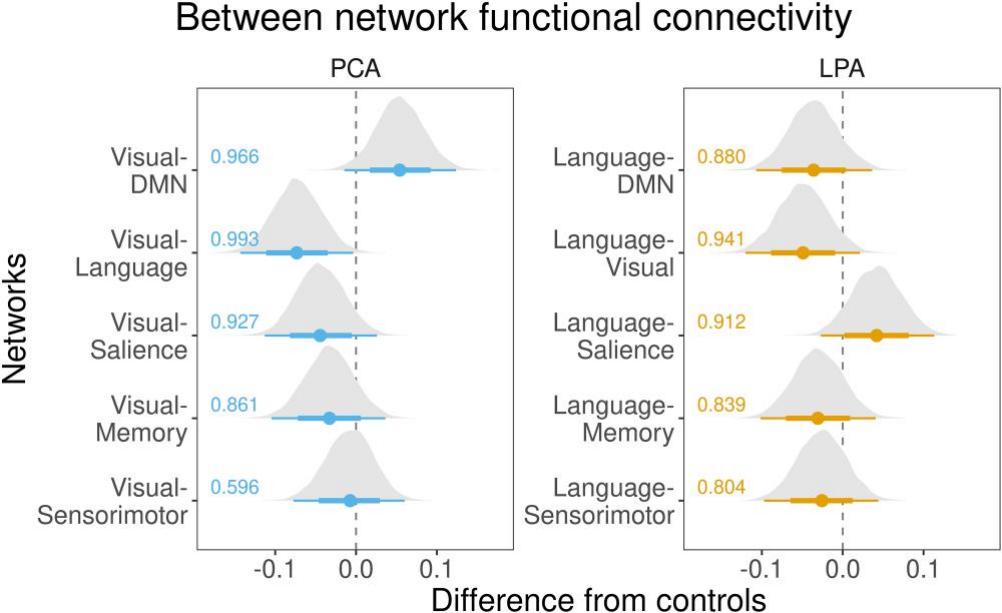
**Between-network functional connectivity.** These forest plots compare between-network connectivity for the six major networks, from the visual network in PCA and the language network in LPA from controls. The plot shows estimates (median), 80% posterior interval (thick line) and 98% posterior interval (thin line). It is interpreted as follows: when the 80% posterior interval does not touch zero, we say there is moderate evidence (Pb > 0.90) of difference, and when the 98% posterior interval does not touch zero, we say there is strong evidence (Pb > 0.99) of difference from controls.

**Figure 7:**
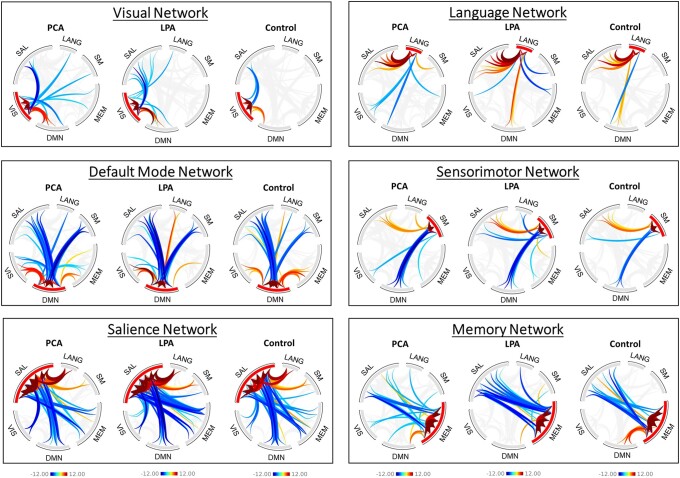
**Functional connectivity network-level group maps.** Group-level (*P* < 0.001, FDR; cluster threshold = *P* < 0.05, FDR) functional connectivity patterns for the six networks are shown here for PCA, LPA and controls. The plot shows *t*-scores generated from one-sample *t*-tests. Abbreviations: DMN = default mode network; LANG = language; MEM = memory; SAL = salience; SM = sensorimotor; VIS = visual.

**Figure 8:**
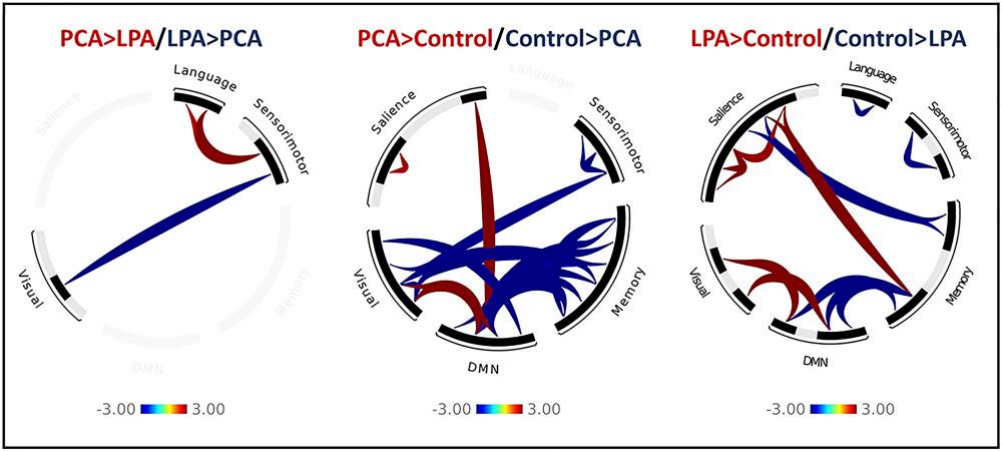
**Functional connectivity network-level difference maps.** Functional connectivity difference (*P* = 0.01, cluster size = FWE 0.05) maps between PCA and LPA and from controls. The plot shows *t*-scores generated from two-sample *t*-tests.

The between-network BHLM for the visual network in PCA showed evidence of reduced visual-to-language (Pb > 0.993) connectivity, with reduced visual-to-salience (Pb > 0.927) connectivity, compared to controls (Fig. 6). An increase in visual-to-DMN (Pb > 0.966) connectivity compared to controls was observed (Fig. 6). At the network level (Fig. 7), individual group maps represent correlations in yellow-to-red connections and anticorrelations in blue connections, while group difference maps (Fig. 8) represent an increase in connectivity with yellow-to-red connections and a decrease in connectivity with blue connections. Both PCA and LPA showed an increase in visual-to-DMN network connectivity, with PCA also showing a significant decrease in visual-to-memory and visual-to-sensorimotor network connectivity compared to controls. PCA also showed a significant decrease in visual-to-sensorimotor connectivity compared to LPA (Fig. 8).

The between-network BHLM for the language network in LPA showed evidence of reduced language-to-visual (Pb > 0.941) connectivity and an increase in language-to-salience (Pb > 0.912) connectivity compared to controls (Fig. 6). At the network level, both PCA and LPA did not differ significantly from controls, but PCA showed an increase in language-to-sensorimotor connectivity compared to LPA (Fig. 8).

Other between-network differences were identified at the network level. Both PCA and LPA showed reduced DMN-to-memory connectivity with an increase in DMN-to-salience connectivity for PCA and memory-to-salience connectivity for LPA (Fig. 8). The effect of adjustment factors is shown in Supplementary Fig. 2.

### Associations between functional connectivity and clinical tests

**Figure 9:**
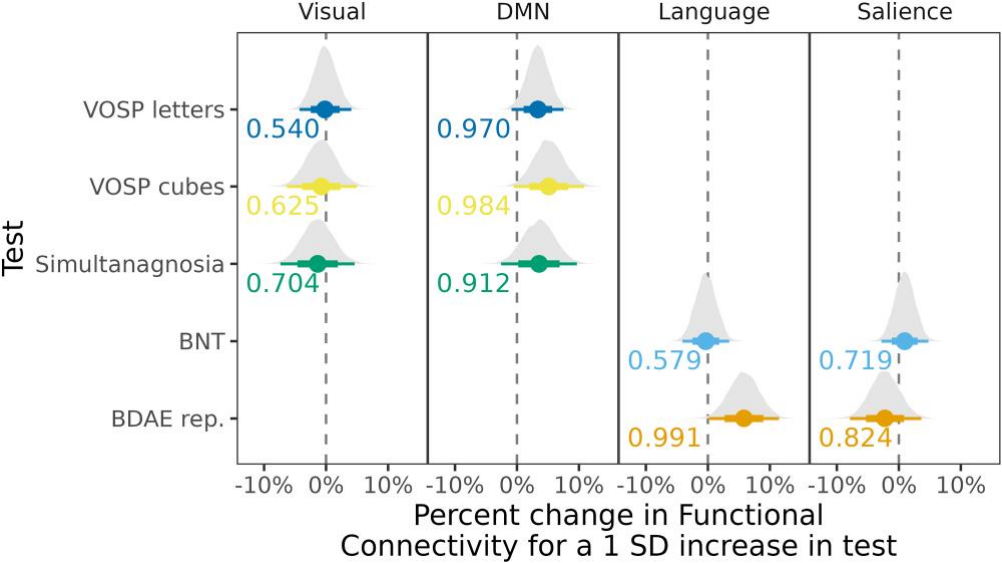
**Associations between clinical scores and within-network connectivity.** This plot shows the associations for the visual tests in PCA and language tests in LPA with their dominant networks. The plot shows estimates (median), 80% posterior interval (thick line) and 98% posterior interval (thin line). It is interpreted as follows: when the 80% posterior interval does not touch zero, we say there is moderate evidence (Pb > 0.90) of difference, and when the 98% posterior interval does not touch zero, we say there is strong evidence (Pb > 0.99) of difference from controls.

The BHLMs assessing associations between within-network connectivity and clinical performance (Fig. 9) showed evidence for a positive association between DMN within-network connectivity and VOSP letters (Pb > 0.967), VOSP cubes (Pb > 0.984) and simultanagnosia (Pb > 0.912) in PCA. No relationships were observed between these three clinical tests and visual network connectivity. In LPA, there was evidence for a positive association between language within-network connectivity and BDAE repetition (Pb > 0.991), with no significant associations observed for BNT (Fig. 9). The effect of adjustment factors is shown in Supplementary Fig. 3.

### Sensitivity analysis

The discriminatory power of functional connectivity compared to grey matter volume and tau-PET uptake was also investigated, and overall, both metrics performed better than functional connectivity in discriminating PCA and LPA from controls and each other, particularly in the visual regions for PCA and language regions for LPA, with visual regions offering better discriminatory power for differentiating PCA from LPA (Supplementary Table 1).

In PCA, visual and DMN grey matter volumes showed a positive association with VOSP letters, with visual grey matter volumes showing associations with VOSP cubes and simultanagnosia. Visual tau-PET uptake showed a negative association with all three clinical tests and no associations with DMN tau-PET uptake (Supplementary Fig. 4). In LPA, language and salience grey matter volumes showed a positive association with BNT, with language grey matter volumes showing associations with BDAE repetition also. No associations between tau-PET uptake and clinical performance for either network were observed (Supplementary Fig. 5).

## Discussion

This study investigated functional connectivity networks in PCA and LPA. We demonstrate disrupted within-network connectivity across many brain networks, particularly in the visual network for PCA and the language network for LPA. Additionally, we demonstrate altered functional connectivity between multiple networks, specifically with evidence of increases in visual-to-DMN connectivity in PCA and language-to-salience connectivity in LPA. These differences in the affected dominant network and their heightened crosstalk with certain networks may be indicative of brain network reorganization occurring in these AD phenotypes.

The visual network included ROIs in the primary visual cortex and the visual association area in the occipital lobe.^61^ Consistent with the clinical presentation and patterns of neurodegeneration observed in PCA,^62^ the PCA patients showed reduced connectivity within occipital regions of the visual network compared to both LPA and controls. This finding also concurs with previous studies that have similarly found visual network disruptions in PCA.^27,32^ We extend these findings by showing that not only do PCA patients have reduced connectivity within the visual network, but they have altered connectivity between the visual network and other brain networks. Reduced connectivity was observed between the visual and language networks, seen in the form of anticorrelations, which could be manifesting in the form of language impairment that typically develops in PCA patients.^12,63^ However, increased connectivity was observed with the DMN, perhaps suggesting that reductions within the visual network prompt increased crosstalk with the DMN.

Therefore, an interesting finding was the increase in connectivity between the visual network and the DMN. This occurs even though within-network connectivity was also reduced in the DMN in PCA. It is important to note that the visual network is correlated to the DMN in controls, but in PCA, it shows both correlations and anticorrelations to different ROIs within the DMN. Therefore, the overall increase in functional connectivity or heightened crosstalk between the two networks is a representation of the functional connectivity of the network, not the individual ROIs within that network. Previous studies have similarly found reduced DMN connectivity in atypical AD phenotypes.^64^ This heightened between-network connectivity may indicate a compensatory mechanism to help preserve cognitive function, specifically non-visuospatial cognitive functions. Similar compensatory mechanisms have been reported in cognitively unimpaired patients^65^ and are being explored in typical AD.^66,67^ Another explanation for this heightened between-network connectivity could be the cascading network failure hypothesis that proposes the initial collapse of the posterior DMN, which eventually leads to higher connectivity between posterior DMN and other connectivity hubs in the brain.^25^ We also found evidence of reduced connectivity between the visual and salience networks, along with a tendency for increased connectivity within the salience network in PCA. These findings fit with the fact that salience network connectivity increases as DMN connectivity decreases in typical amnestic AD^24,68,69^ and suggest that these network dynamics also occur in PCA. We also observed decreased DMN connectivity and increased salience network connectivity in LPA. Hence, it is possible that these network alterations could be a characteristic feature of AD regardless of the clinical presentation.

The language network included the two main language centres, Broca’s area in the inferior frontal gyrus and Wernicke’s area in the posterior superior temporal gyrus.^70^ Our findings showed reduced connectivity within left temporal regions of the language network in both PCA and LPA, which was worse in LPA. Further, LPA showed evidence of reduced language network functional connectivity compared to both PCA and controls. These findings are concordant with current studies reporting associations between language deficits and selective atrophy and network disruptions within the language centres in LPA.^28^ As discussed above, language deficits can also develop in PCA patients.^12,63^ Disrupted connectivity within the language network in LPA may instigate brain network compensation and reorganization by making new connections with other networks. Indeed, new connections with the salience network were noted in LPA. We observed reductions in connectivity between the language and visual networks, which could portend towards the development of visuospatial difficulties in LPA.^71,72^ The finding that both LPA and PCA showed reductions in connectivity between these two networks may reflect the possibility of clinical overlap often occurring between these AD phenotypes. It is possible that reductions in connectivity between these networks lead to the development of overlapping clinical features.

Another important finding was the increase in connectivity between the language and salience networks, along with a slight trend for an increase in within-salience network connectivity in LPA. These findings may reflect towards the importance of inferior parietal involvement in LPA networks, particularly in the form of increased salience network connectivity, which might result in an oversensitivity to salient events, such as irritability and anxiety in LPA patients.^73,74^ These findings are in line with reports from typical AD patients, where enhanced salience network connectivity^24^ led to heightened emotional sensitivity,^75^ while remaining attuned to non-verbal emotional communication and cues.^76,77^

Reduced within-network connectivity was also observed in the sensorimotor network in both PCA and LPA. These findings may indicate an early decline in atypical AD phenotypes with the possibility of network reorganization involving the sensorimotor network. The only network that did not show reduced within-network connectivity in PCA and LPA was the memory network. This network involved the amygdala, hippocampus and anterior parahippocampal gyrus. These findings concur with previous reports showing relative preservation of the medial temporal lobe in PCA^13^ and LPA.^17,78^

Within PCA, clinical scores for simultanagnosia, VOSP letters and VOSP cubes were not associated with within-network connectivity in the visual network. The absence of such an association could reflect involvement of other brain regions outside of the purely occipital visual network. Imaging studies on PCA have reported the presence of PCA subgroups, with atrophy, hypometabolism and tau uptake mainly in the parieto-occipital cortices (dorsal), temporo-occipital cortices (ventral) and primary occipital cortex (primary occipital).^5,79^ Our results showed evidence of a positive correlation between DMN within-network connectivity and scores for simultanagnosia, VOSP letters and VOSP cubes, which could indicate involvement of the parietal cortex. Previous studies have found associations between parietal lobe atrophy and simultanagnosia^80^ and the VOSP,^81^ suggesting that the involvement of the parietal cortex could be important for these PCA features.

Regarding the two language centres, atrophy in Wernicke’s area shows that correlates with repetition performance^82,83^ and deficits in the phonological loop^28^ are a core mechanism in LPA,^84^ whereas atrophy in Broca’s area is generally associated with agrammatic aphasia, which is absent in LPA. Our data showed evidence of a positive correlation between within-network connectivity in the language network and BDAE repetition scores, consistent with findings of repetition deficits in LPA. However, we did not find any evidence of associations between within-network connectivity in the language network and BNT scores. The absence of an association with confrontation naming could suggest a lack of sensitivity in detecting focal associations, for instance, between the temporal pole and BNT scores.^85^ It could also be due to the involvement of regions outside the language network.

Although our sensitivity analysis showed that both grey matter volume and tau-PET uptake offered better discriminatory power than functional connectivity and showed multiple associations with clinical performance, functional connectivity investigations offer several advantages and help further our knowledge on the neurobiology of PCA and LPA. The fact that these syndromes are associated with so many changes in connectivity between different brain networks suggests that the disease mechanisms underlying atypical AD have a widespread influence throughout the brain, a fact that needs to be considered when designing management strategies and treatment approaches. An advantage of rsfMRI over traditional volumetric or diffusion techniques that measure neurodegeneration is the ability to assess network stability, identifying both reduced and increased network communication and providing a more fine-grained evaluation of the effect of neurodegeneration on brain mechanisms. The within-network and between-network connectivity metrics we identified could potentially be translated into diagnostic biomarkers that would be able to segregate healthy from disease states and differentiate atypical AD phenotypes. One important finding of this study was relating within-network connectivity to specific clinical tests, which allows for the identification of network abnormalities that may influence clinical outcomes. This knowledge could lead to the identification of targets for treatment and could help guide better management strategies in atypical AD patients. Future longitudinal studies could also offer the potential to study clinical progression and the value of rsfMRI as a prognostic biomarker. Together, the findings from this study offer promise for improving clinical applicability of rsfMRI techniques.

Strengths of this study include the large PCA and LPA cohorts, the consistent and comprehensive clinical evaluations and the fact that our models controlled for differences in age, sex and education. Our findings call attention to the stark differences in connectivity seen within and between multiple networks, which could act as potential markers for differentiating atypical AD phenotypes from controls. A potential limitation is the absence of other less common clinical phenotypes of atypical AD including dysexecutive, behavioural and corticobasal presentations.^3^ Future studies including a more heterogeneous cohort and longitudinal data will be needed to assess how these network changes evolve based on diagnosis and over time.

## Conclusion

Our results characterize the disruptions in functional connectivity in both PCA and LPA, with phenotype-specific reductions within the dominant networks, visual for PCA and language for LPA. However, evidence for heightened crosstalk between the visual network and DMN in PCA and language and salience networks in LPA was also observed, which suggests possible brain network reorganization possibly following impairment of phenotype-specific dominant networks. Together, these findings contribute to our understanding of brain network disruptions and reorganization in atypical AD and suggest that phenotype-specific within- and between-network connectivity could be a potential marker for differentiating PCA and LPA from controls.

## Supplementary Material

fcad184_Supplementary_Data

## Data Availability

The data that support the findings of this study will be available from the corresponding author on request.

## Abbreviations

AD =: Alzheimer’s disease
AVLT-RPC =: Rey Auditory Verbal Learning Test—Recognition Percent Correct
BDAE =: Boston Diagnostic Aphasia Exam
BHLM =: Bayesian hierarchical linear model
BNT =: 15-item Boston Naming Test
DMN =: default mode network
LPA =: logopenic progressive aphasia
MOANS =: Mayo Older Americans Normative Studies
MoCA =: Montreal Cognitive Assessment Battery
MPRAGE =: magnetization-prepared rapid gradient echo
NPI-Q =: Neuro-Psychiatric Inventory Questionnaire
Pb =: posterior probability
PCA =: posterior cortical atrophy
Rey-O =: Rey-Osterrieth Complex Figure Test
ROIs =: regions of interest
UPDRS III =: Unified Parkinson’s disease rating scale III
VOSP =: Visual Object and Space Perception Battery
