# Supplementary material for: Altered within- and between-network functional connectivity in atypical Alzheimer’s disease
Source: Brain Commun. 2023 Jun 14;5(4):fcad184. doi: 10.1093/braincomms/fcad184 (PMC10331277; doi:10.1093/braincomms/fcad184)
Supplement: fcad184_Supplementary_Data [file fcad184_supplementary_data.pdf]

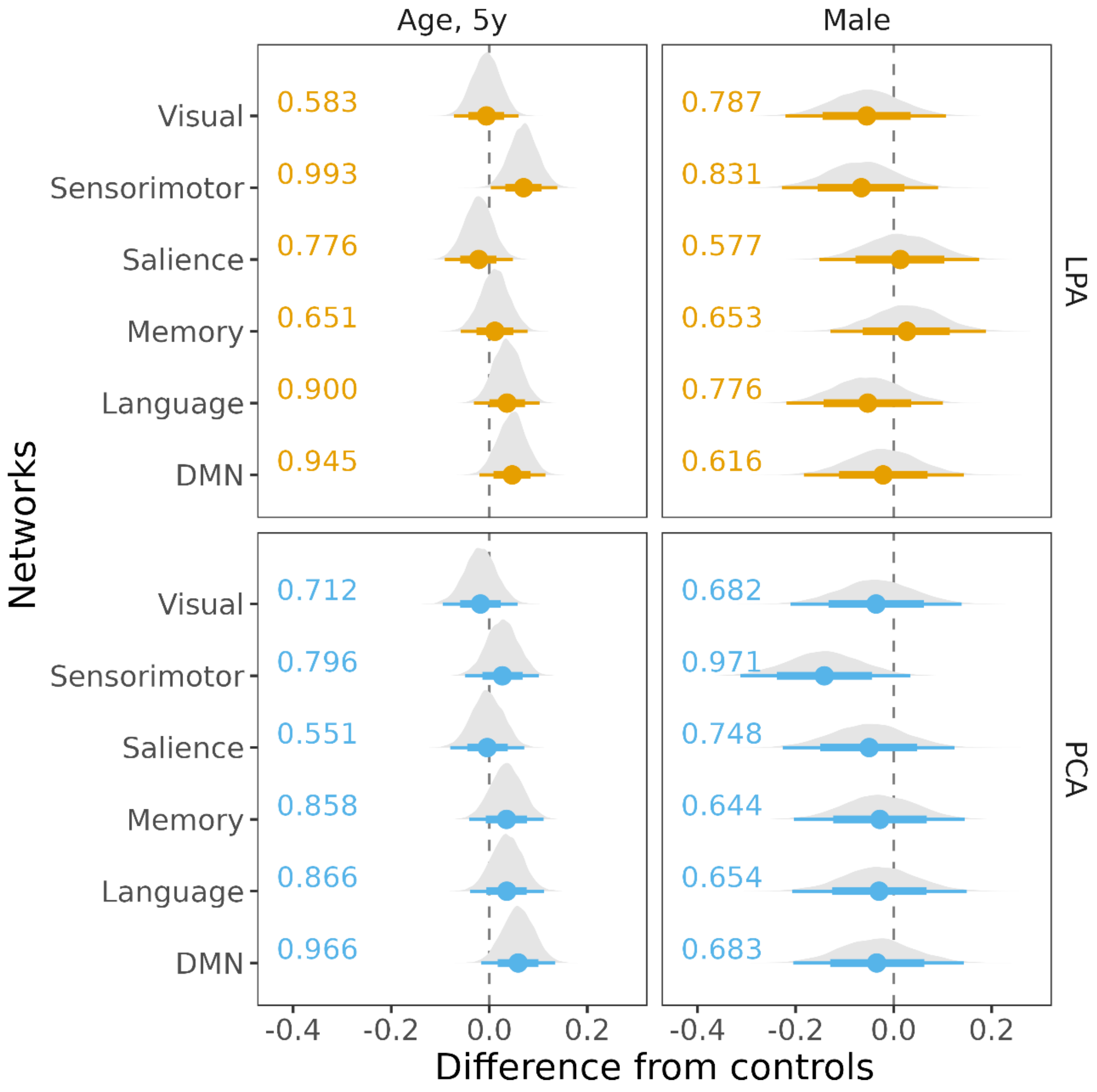

Supp figure 1: The BHLM model was adjusted for covariates of age and sex to quantify within-network connectivity in PCA and LPA. The plot shows estimates (median), 80% posterior interval (thick line) and 98% posterior interval (thin line). It is interpreted as follows; when the 80% posterior interval does not touch zero, we say there is moderate evidence (Pb>0.90) of difference and when the 98% posterior interval does not touch zero, we say there is strong evidence (Pb>0.99) of difference from controls.

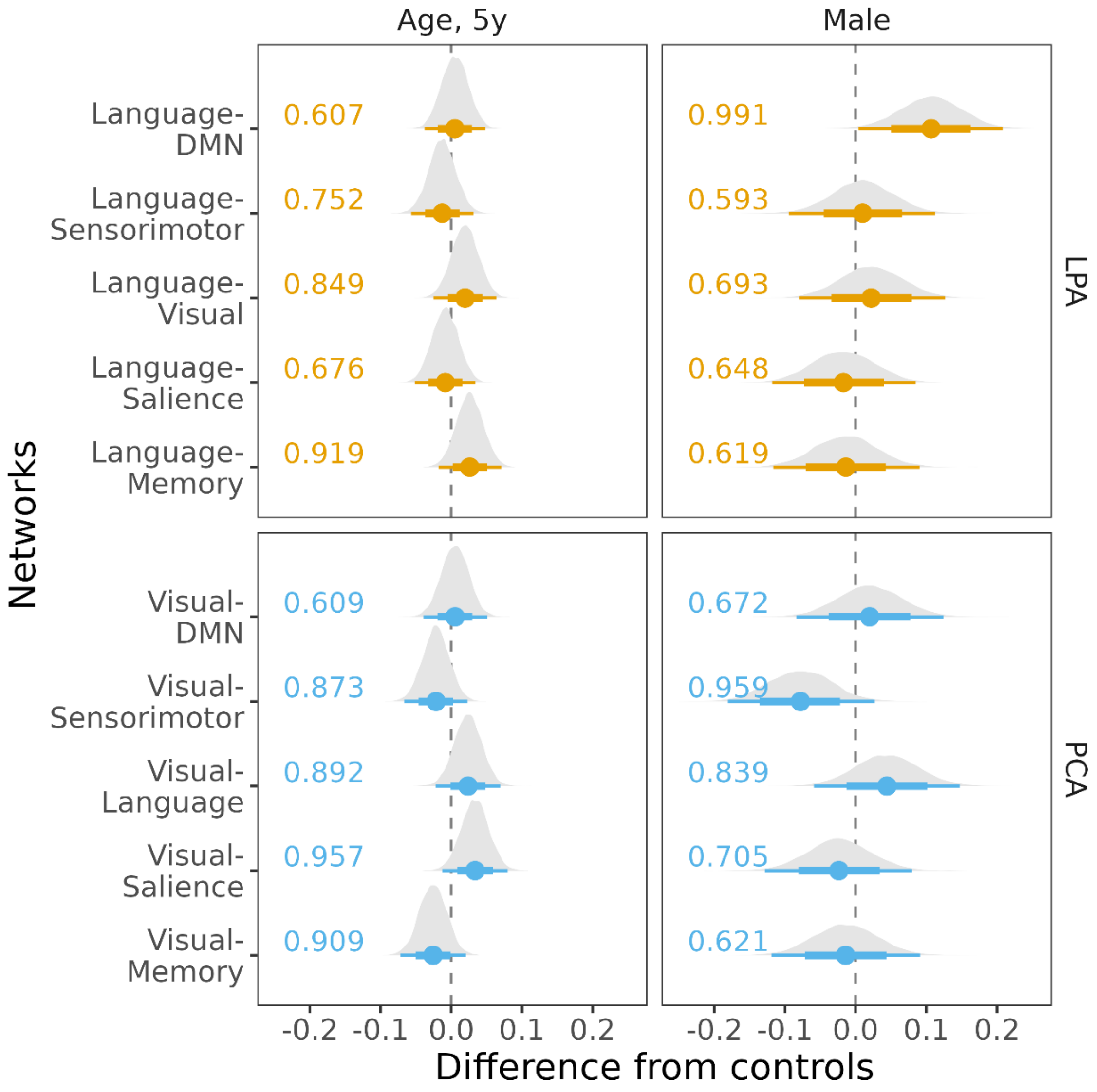

Supp figure 2: The BHLM model was adjusted for covariates of age and sex to quantify between-network connectivity for the visual network to all other networks in PCA and for the language network to all other networks in LPA. The plot shows estimates (median), 80% posterior interval (thick line) and 98% posterior interval (thin line). It is interpreted as follows; when the 80% posterior interval does not touch zero, we say there is moderate evidence (Pb>0.90) of difference and when the 98% posterior interval does not touch zero, we say there is strong evidence (Pb>0.99) of difference from controls.

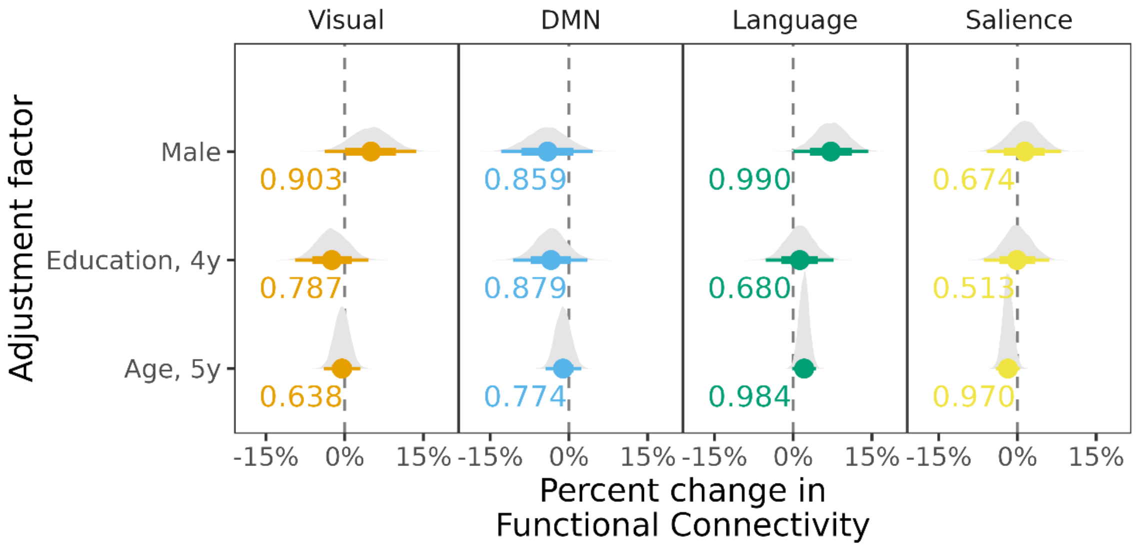

Supp figure 3: The BHLM model was adjusted for covariates of age, sex and education to quantify relationships between performance on clinical measures and within-network connectivity. The plot shows estimates (median), 80% posterior interval (thick line) and 98% posterior interval (thin line). It is interpreted as follows; when the 80% posterior interval does not touch zero, we say there is moderate evidence (Pb>0.90) of difference and when the 98% posterior interval does not touch zero, we say there is strong evidence (Pb>0.99) of difference from controls.

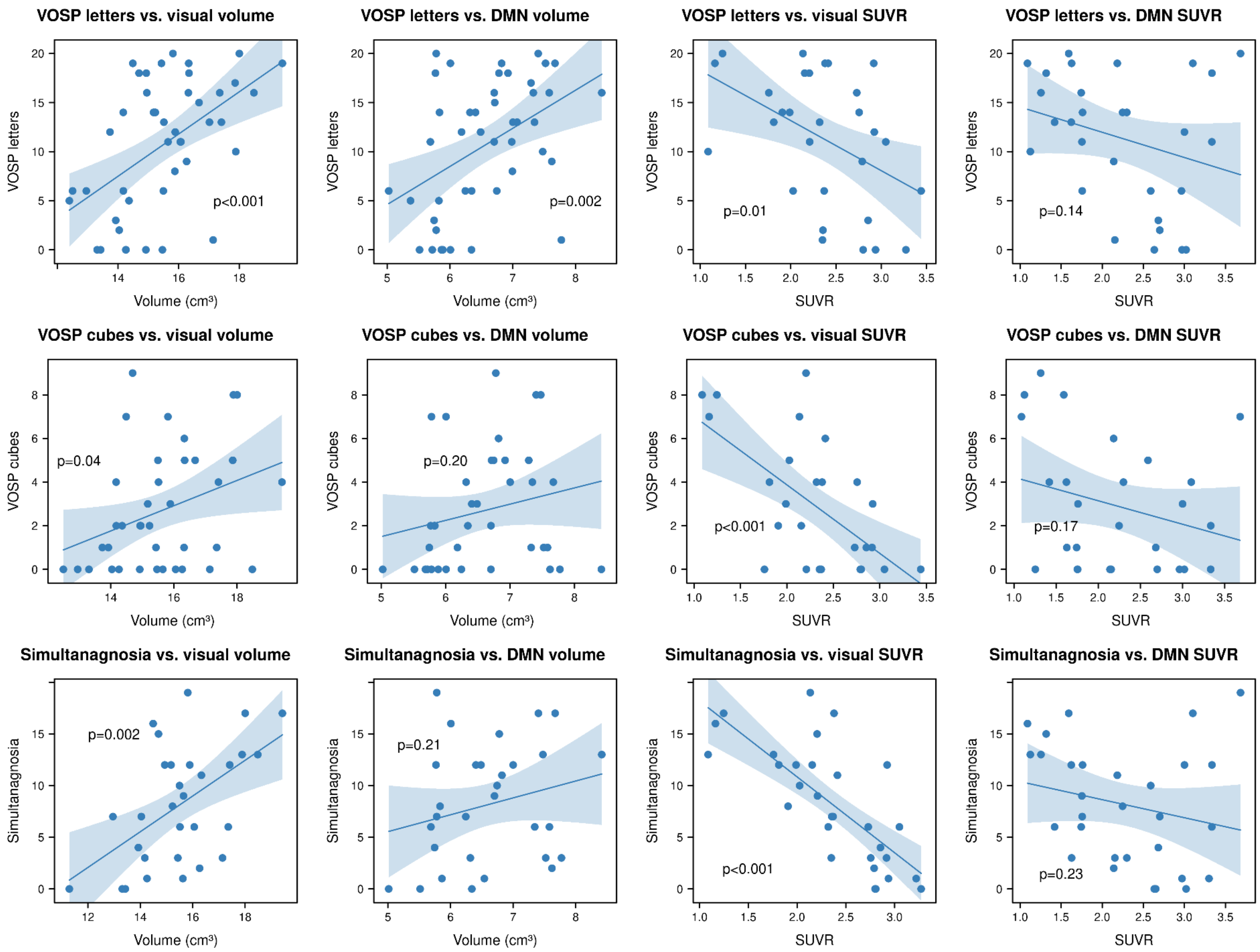
VOSP letters vs. visual volume
VOSP letters vs. DMN volume
VOSP letters vs. visual SUVR
VOSP letters vs. DMN SUVR
VOSP letters
Volume (cm³)
SUVR
p<0.001
p=0.002
p=0.01
p=0.14
VOSP cubes vs. visual volume
VOSP cubes vs. DMN volume
VOSP cubes vs. visual SUVR
VOSP cubes vs. DMN SUVR
VOSP cubes
p=0.04
p=0.20
p<0.001
p=0.17
Simultanagnosia vs. visual volume
Simultanagnosia vs. DMN volume
Simultanagnosia vs. visual SUVR
Simultanagnosia vs. DMN SUVR
Simultanagnosia
p=0.002
p=0.21
p<0.001
p=0.23

Supp figure 4: Linear regression model to quantify relationships between performance on clinical measures and neuroimaging metrics of gray matter volume and tau-PET SUVRs in PCA.

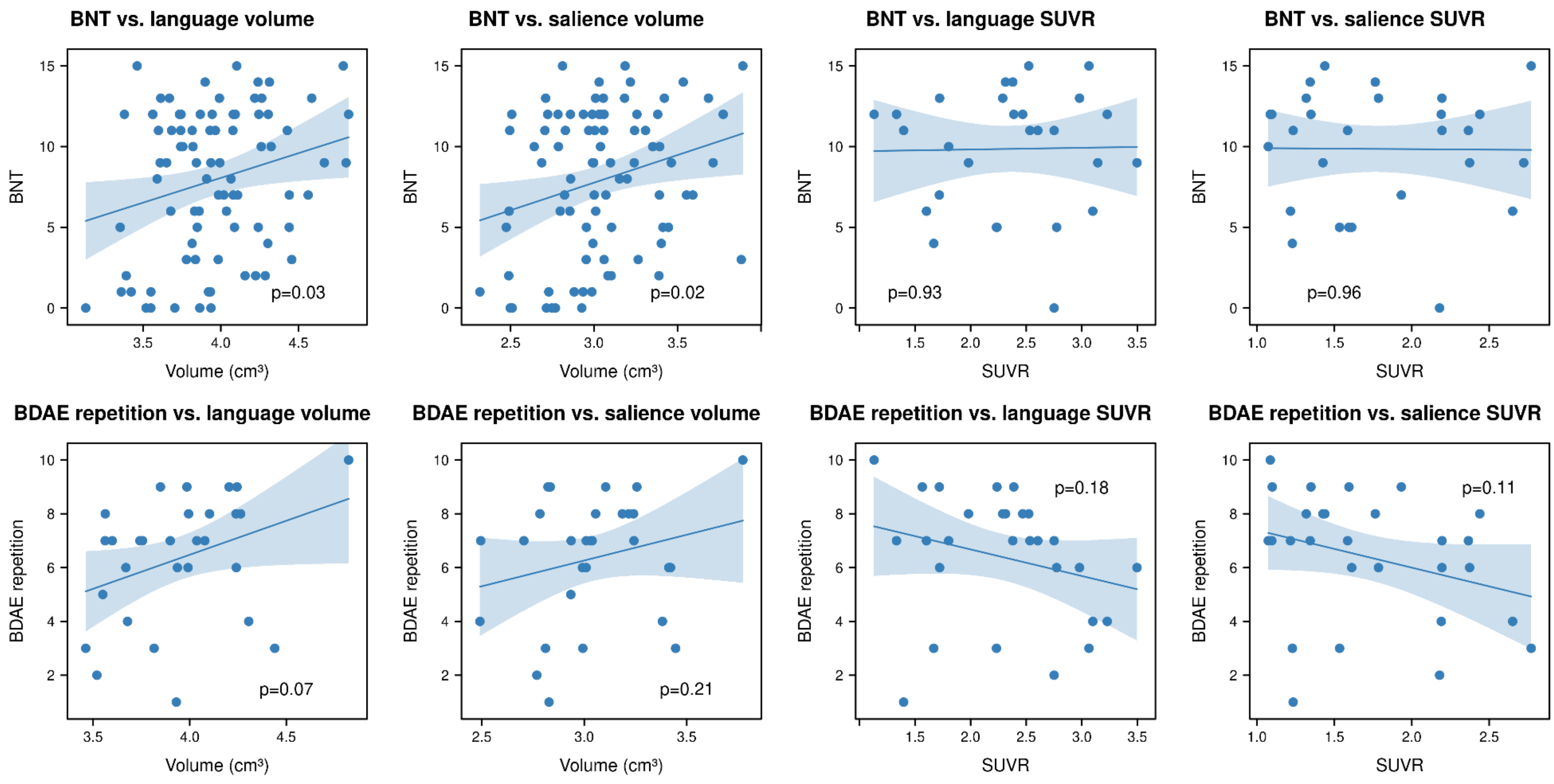

Supp figure 5: Linear regression model to quantify relationships between performance on clinical measures and neuroimaging metrics of gray matter volume and tau-PET SUVRs in LPA.

Supp Table 1. AUROC scores for functional connectivity, gray matter volumes and tau-PET uptake for group differences between PCA, LPA and controls

	**Network**	**Functional connectivity**	***Gray matter volumes***	***Tau-PET***
**PCA > Controls**	Visual	0.61	*0.97*	*0.96*
	Language	0.56	*0.93*	*0.94*
	DMN	0.61	*0.95*	*0.93*
	Memory	0.56	*0.91*	*0.95*
	Salience	0.50	*0.83*	*0.88*
	Sensorimotor	0.62	*0.92*	*0.85*
**LPA > CU**	Visual	0.56	*0.85*	*0.90*
	Language	0.68	*0.92*	*0.97*
	DMN	0.57	*0.88*	*0.94*
	Memory	0.51	*0.90*	*0.93*
	Salience	0.59	*0.82*	*0.91*
	Sensorimotor	0.55	*0.88*	*0.85*
**PCA > LPA**	Visual	0.53	*0.86*	*0.83*
	Language	0.60	*0.60*	*0.55*
	DMN	0.51	*0.72*	*0.61*
	Memory	0.57	*0.52*	*0.77*
	Salience	0.58	*0.52*	*0.55*
	Sensorimotor	0.55	*0.62*	*0.56*

Scores above 0.80 are considered as good AUROC scores, while above 0.90 are considered as excellent AUROC scores.
